# Selections

**Published:** 1860-11

**Authors:** 


### Selections.

### ANÆSTHETICS IN HOSPITAL PRACTICE.

The discovery of anæsthetics was universally hailed as a great and unqualified blessing to man, the victim of unavoidable pain. The year of the announcement of the power of ether to render the patient insensible under the hand of the operator, was distinguished as the *annus mirabilis;* it began a new era in the history of operative surgery, and the older surgeon, in the language of the elder Warren, "wished again to go through his career under the new auspices." We can well imagine with what enthusiasm he who had been accustomed to struggle through difficult operations on patients forcibly held, now pursued his dissections as on the cadaver, and saw the patient, on the completion of the operation, suddenly restored to a full possession of all his faculties, as if by magic. And when, a year or more after these first experiments with ether, chloroform was introduced to notice, so agreeable to the senses, so prompt in its action, and so harmless in its effects, the perfection of anæsthetic agencies was thought to have been attained.

But every good must have its corresponding ill. It was soon announced that a lady, sitting in a dentist's chair, had suddenly expired while inhaling chloroform preparatory to the extraction of a tooth. A second, third and fourth case was reported, and always previously to some trivial operation. The faith of its friends, however, remained unshaken, and these unfortunate results were attributed to the attending circumstances, and not to the anæsthetic. At length fatal cases began to occur occasionally in hospitals, in the presence of eminent physicians and surgeons, and in spite of their previous precautions, and efforts to resuscitate the victim. Finally the fact seemed established beyond peradventure, that chloroform is not an innocuous agent even under circumstances apparently the most favorable for its administration, by the occurrence of a fatal case (in a dentist's chair, however,) in spite of the persistent and well directed efforts of Professor Simpson himself to restore animation.

It can now no longer be denied that anæsthetics are followed by unpleasant and occasionally fatal effects in a given number of instances. The latest statistics that have been published are as follows:—Total fatal cases in Europe, one hundred and twenty-five. When we take into account the aggregate cases of anæsthetization during the last sixteen or seventeen years, of their almost universal use in hospitals, and in private practice, this mortality is a percentage of the whole number of cases, positively infinitesimal. It is doubtful if any active remedy of the materia medica can show a better record.

The recent death by chloroform in Bellevue Hospital has, we understand, raised the question in the Medical Board as to the propriety of allowing this agent to be longer employed for purposes of anæsthesia in that institution. Before this question can be properly decided, the comparative merits of ether and chloroform must be considered, for anæsthetics in some form are now indispensable to the practice of operative surgery and midwifery, and can never be discarded, even though the mortality from their use were tenfold its present per centage. And before chloroform is stricken from the list, it were well to inquire as to the real sources of danger from its use, for if it is demonstrated that under circumstances it is as safe as any anæsthetic, every surgeon will, under such circumstances, prefer chloroform.

The comparative merits of ether and chloroform, as anæsthetics, it is not easy to decide. The statistics which we have given above show, that of the one hundred and twenty-five fatal cases from anæsthetics in Europe, twenty-five occurred during the inhalation of ether, and one hundred of chloroform, giving a mortality from the latter equal to four-fifths of all the cases. Although chloroform would seem by this exhibit to be the more fatal anæsthetic, yet a moment's reflection will convince any one that it may not even approximate the truth, for we have no knowledge of the per-centage of the deaths to the number of cases of administration of either agent. It might, and probably would appear, could we sift this subject thoroughly, that chloroform had been given four times as often as ether during that period. We may, however, arrive at a very satisfactiory conclusion as to the safety of chloroform, by taking the gross number of cases of its administration in certain well authenticated instances, and noting the results. For example, it was given twenty-five thousand times by the French, in the Crimean war, without a single fatal issue. It is freely used in midwifery by many eminent English and American obstetricians, and, we believe, no fatal case has yet been reported in this department of practice. Professor Simpson is stated to have used from five to seven gallons annually for thirteen years, without an unfavorable result.

The real sources of danger in the employment of chloroform have not been sufficiently studied. Authors mention:—1st, *A full stomach;* for vomiting being a common symptom in chloroform inhalation, the patient is liable to be suffocated. 2d, Affections of the nervous system, as delirium tremens, epilepsy, hysteria, etc. 3d, Affections of the vascular system, as fatty degeneration of the heart, atheromateous deposits, etc., etc. We do not propose to discuss these contra-indications to the use of chloraform, as it is by no means as yet established how far these conditions are to be regarded as complicating its effects. We believe, however, that it was maintained by the late Dr. Snow, whose opinion on all subjects relating to chloroform is entitled to our confidence, that even when lesions of the nervous and vascular systems do exist, chloroform properly administered, is far less dangerous than an operation without an anæsthetic From some recent investigations as to the nature of death from chloroform, the following interesting facts appear:—1st, That the great majority of deaths (two-thirds) occur in slight operations, as those performed on sphineters, tendinous sheaths, strabismus, tooth-drawing, etc., but few during the larger operations, as amputations, resections, ovariotomy, etc. 2d, In the majority of fatal cases by chloroform, death occurred before the operation,—during the first stage of inhalation—the stage of excitement. 3d, That the deaths that have occurred after the operation, and were attributable to the anæsthetic, have generally been when ether was slowly administered, or ether and chloroform, but not pure chloroform. Without dwelling on these subjects, which are all of the deepest interest to those who are discussing the question of the relative or actual merits of the different anæsthetics, we shall allude to what we consider if not the real, certainly the great source of danger in the use of anæsthetics in general, and chloroform in particular, in our hospitals we refer to the gross and culpable carelessness of their administration. Rarely is the patient carefully examined by a competent person to determine if there be any contra-indication to the use of anæsthetics—a point that should never be neglected. The delicate and most responsible task of administering the agent is usually committed to a junior physician, who has no knowledge whatever of the nature of his duties ; he knows nothing of the different stages through which the patient is to pass, or of the value of the symptoms which appear during the administration ; his inhaler is a towel well saturated, and his directions often are to apply it directly to the face. The stage of *profound coma* having been reached, the operator seizes the scalpel, and all eyes are directed to its movements; the innocent junior, all absorbed in the operation, forgets his duty, unconsciously drops the towel upon the patient's face, and occasionally adds the weight of his body to its suffocating effect, as he leans forward in the anxious pursuit of knowledge. At length a moan, or the collapse of the jetting arteries, or the suggestion of a bystander more interested in the sufferer than the operation, recalls attention to the condition of the patient. Naturally enough he has ceased to breathe; the operation is suspended; the messenger is dispatched for brandy; and in the meantime artificial respiration by the most approved method is attempted by every available means. Fortunately the patient is generally resuscitated, at least sufficiently to have the operation completed, and be taken to the ward.

We do not here give an overdrawn picture, for such scenes if haply not more unpleasant, may be witnessed in our hospitals almost weekly. The reform should commence with the mode of administration of these agents. A medical man of known ability should be selected to administer the anæsthetic; we say medical man, because he will not become so interested in the operation as to forget his duties. To his care should be committed, so far as practicable, every patient who is about to submit to an operation. This is but that precaution which every surgeon exercises in private practice, and hence the few cases of death from anaesthetics which occur outside of our hospitals. If this degree of care is exercised in our hospitals and still fatal consequences follow the use of ether or chloroform, or both, the question may well be raised as to the propriety of rejecting the more dangerous.—*Am. Med. Times.*

### A RARE FORM OF FRACTURE OF THE LOWER JAW TREATED BY A NOVEL METHOD.

BY DR. E. J. FOUNTAIN.

In the spring of 1856, W. G——, æt. 42, fell from a height about ten feet, striking his chin upon a hard piece of timber. The result was a fracture of the lower jaw through the body on either side, and also through the neck of the condyle on the left.

The deformity was characteristic of fracture of the neck—namely, a displacement of the whole of the lower jaw backwards and latterally to the side of the fracture. The middle portion was disconnected by simple fractures without displacement, around which the membranous and muscular tissues held sufficiently firm to admit of sufficient traction upon the part posterior to the middle fragment by force applied to the latter in the region of the symphysis. By careful manipulation, crepitus could be felt at each point. Upon releasing the traction in front, the deformity immediately returned, by which it became evident that the fracture through the neck was oblique, permitting the retractive force of the muscles to reproduce the deformity as soon as the force which held it in front was removed. It became evident at once that to retain this fractured portion permanently in apposition so as to prevent deformity, would be the greatest difficulty. A temporary application of the pasteboard splint and four-tailed bandage was applied, and ordinary measures used to guard against excessive inflammation. During the following eight days, I tried every form of dressing by splints and bandaging that my ingenuity could devise to effect a *permanent* reduction of the fracture of the neck, and in this I was assisted by two skillful surgeons, Drs. O'Reardon and Adler, but all to no purpose. The middle fracture gave me no trouble but in arranging the fractured neck, I could derive no aid from my friends, and found no light to guide me by consulting our text books on surgery. There was no difficulty in properly reducing the fracture, but after laboring for hours with all manner of appliances to retain it in place, I would invariably find the chin slipping back, and to one side, until in a very short time the original deformity would be entirely restored and our united efforts completely baffled. On the eighth day after the injury we all labored for several hours to secure a correct and permanent position of the parts, and had finally to abandon all our efforts as ineffectual. The case was now a truly distressing one. The family became impressed with the belief that it could never be accomplished, and the prospect of a serious and unsightly deformity for life created an anxiety and grief which can hardly be described.

A method then occurred to me which I at once announced as a *sure* way and the *only* way by which deformity might be prevented, and the parts made to unite permanently in their true position. The jaw was displaced backwards and laterally towards the injured side. By firm and steady traction it could be brought out in place, and now the question was *how* to retain it there. It became evident to me that some unvarying, persistent force must be applied to a fixed point in such a manner as to take the place of my hand or the instrument used in bringing it out and holding it there. The anterior portion of the superior maxillary, or its prolongation by the teeth, presented this fixed point, and by *drilling holes* in one or more of the upper and lower teeth in front, and firmly *wiring* them together in their natural position, the lower jaw must of necessity be prevented from falling backwards or to one side. Wrapping wire around the teeth could not be depended upon. The holes drilled for this purpose I thought could be easily filled by a dentist and thus preserved; and even though much injured or destroyed, how trifling this loss compared with a lasting deformity of the whole jaw—unsightly in appearance and serious in its effects upon the voice and mastication. I at once obtained the services of a dentist to drill the teeth, which was found to be no very light operation, owing perhaps to a want of proper instruments. In order to make sure of a perfect reduction of *lateral* as well as backward displacement, I had the holes made in such a manner that the traction of the connecting wire would not only hold the two bones firmly together, but also draw the jaw to the side *opposite* the fractured neck, in order that the loop of wire might not, like a link, swing slightly to one side by the muscular contraction. I therefore had a hole drilled directly through a front incisor above, and another through one below, not directly opposite in their natural position, but through the one adjoining it on the one side towards which the bone was inclined to be drawn. Through these I passed a double strand of fine annealed iron wire, such as is used by jewelers, and drawing the lower jaw forward in its place and holding it firmly against the upper, by aid of assistants, I twisted the wires tightly together. The success was complete. There I now had it exactly in place, and as long as the wires should hold there was no possibility of the slightest displacement.

After this was accomplished the other fractures were easily managed. A pasteboard splint was moulded about the whole of the lower jaw, and retained by the usual four-tailed bandage. The patient had no difficulty in taking liquids, which were easily drawn into the patient's mouth between the teeth, and by this means he was nourished for the *four weeks* during which he patiently endured this artificial *lock-jaw.* In about ten days the wires gave way, and I immediately inserted another cord composed of four of the same wires, and this held the jaw securely and immovably fixed until the fractures were all united.

I removed the wires after the jaw had been thus secured for four weeks, and found that perfect union had taken place at all the points of fracture, *without a particle of deformity*, except such as necessarily resulted from the provisional callus. This was in time gradually absorbed, and now, nearly four years since the accident, no one can tell by his appearance, or by an examination, that any fracture had ever taken place.—*New York Journal of Medicine, Jan.* 1860.

---

*Extreme Congenital Deformity—Separation of the Superior Maxillary bones—Want of Proper Development of the Palatine Processes of the same bone and also of the Palate bones, connected with Hare Lip—Removed by* C. C. FIELD, M. D., of Easton, Pa.—About a year since, the wife of R—— B——, Esq., of ———, Penn., gave birth to a child so hideously deformed as to cause great grief to the parents, and to excite the horror and surprise of all who witnessed it. The deformity consisted of an entire separation, at the suture, of the superior maxillary bones. A wide gap also existed in the roof of the mouth, in consequence of a deficient development of the palatine process of the superior maxillary bones, and also a like deficiency in the palate bones, producing a large opening, extending into the nasal cavities, every part of which could be distinctly seen, as well as the anterior part of the fauces. The separated portion of the right superior maxillary bone extended outward, and turning to the right, drew down the nose to such an extent that the nostrils were scarcely perceptible, the nose being almost even with the face, and particularly the left side of it. Connected with this was a hare lip, or what may perhaps be more fully expressed as an entire separation of the upper lip, being continuous with the opening in the maxilla into the nasal cavities. The wide anterior gap, the flattened nose and the separated and contracted upper lip presented a spectacle of the most hideous character, and spread terror among the neighbors, especially the females. The child, from its condition, was of course prevented from nursing. It was therefore fed on milk, introduced through a tube, until it was 4½ months old. I was called upon by Dr. A. C. Smith, under whose care the child was, to suggest something for its relief.

After examining the child—which was healthy but not robust—with the greatest care, it was determined in consultation to attempt to remove the deformity, and place the parts in such a position that it would be able as it increased in age to masticate, swallow, and have all the advantages of speech, all of which, at least to a very great degree, it would never have been capable of without an operation. All things being considered, it was determined to operate upon the child without delay. Dr. Smith had charge of the case, on whose attention subsequent to the operation much of its success depended. Dr. Bowlsby, of Finesville, and Dr. Bergin, of Easton, were present to assist. The child, being properly secured, was held by an assistant. The two halves of the upper lip and the base of the nose were carefully dissected upward, on a line with the malar processes, and so held, when with strong cutting forceps, the deformed portion of the superior maxilla was removed, also the margins of the palatine processes of the two maxillas and of the two palate bones. For the purpose of approximating as much as possible the two maxillas, they were partially separated from their connection with the malar bones, with the forceps. The bleeding from the ends of the divided bones was most profuse, and could not be stopped by the most powerful astringents, when the actual cautery was resorted to, by which it was at once arrested. The bones were then brought as closely as possible in contact, the edges of the separated lip were removed, the parts united with needles and ligatures, and the whole supported by adhesive strips and bandages. Although at its close the child lay prostrate for several hours, the operation proved entirely successful.

The child is, at present writing, perfectly healthy and entirely free from all deformity, its appearance being so changed that no evidence of the former irregularity can be seen. At the time of the operation, in December last, it was 4½ months old; it is now nearly a year, and all that was hoped to result from the operation has been realized. It can masticate, swallow without any artificial aid, and is as forward in speech and correct enunciation as children of its age.

We send you an account of this case not merely in consequence of the great deformity that existed, but for the purpose of communicating at how early a period so fearful an operation may be performed and the benefits which have resulted from the operation, already enumerated.—*Medical and Surgical Reporter.*

### ETHER AND CHLOROFORM.

By Dr. G. HAYWARD, late Professor of Surgery in the Massachusetts Medical College, Boston.

[The profession is scarcely prepared to receive without qualification the opinion that ether should be substituted for chloroform in all cases, to the entire exclusion of the latter. But the author observes that in no instance has there been any alarming or serious symptoms from the use of sulphuric ether, even when used to a great extent, undiluted with air. It does not produce anæsthesia quite so speedily as chloroform.]

There is no doubt in my mind that sulphuric ether should be used as an anæsthetic agent to the entire exclusion of chloroform. It is as efficacious, and I should say without hesitation, after having seen chloroform administered by others in many cases, that ether produces a more complete state of unconscious insensibility. Its effects pass off sooner, and less vomiting, nausea, and headache follow its inhalation. It is as easily administered. All that is required for its administration is a bell-shaped sponge with a cavity large enough to cover the nose and mouth. If the patient breathes it gradually, little or no irritation is produced in the larynx and air-passages, there is but little if any cough or sense of suffocation, nor a distressing or unpleasant symptom of any kind.

There may be some persons to whom the odor of ether is offensive and irritating, but they are comparatively few, and even they can be brought under its influence without any very great annoyance.

The quantity of sulphur'c ether required to produce anæsthesia depends very much on the manner in which it is administered. If the patient is made to inhale it rapidly, and the atmospheric air is to a great extent excluded, a small amount will be sufficient. From four to eight ounces may be regarded as the average quantity. It is rare to meet with a case in which less than four ounces will be used; and in protracted operations, in which it is desirable to keep up the state of insensibility for a length of time, I have often given more than eight ounces. The ether should at first be poured on the concave part of the sponge; one or two ounces will be enough for this purpose. When the inhalation is going on, it is better to pour the ether on the outside of the sponge so as to avoid the necessity of removing it from the face. From half an ounce to an ounce should be used at a time in this way, till anæsthesia is produced. When this takes place, the patient is wholly unconscious, and has no control over the voluntary muscles. He is unable to raise his eyelids when told to do so, and gives no indication of hearing or consciousness, if spoken to in a loud tone. The pulse usually becomes slower than the ordinary standard, though at the beginning of the inhalation it is quicker.

It is, I am confident, a perfectly safe anæsthetic agent. I have not been able to find any well-attested case of death from its inhalation. There may have been such, but they have never come to my knowledge, though I have taken unwearied pains to obtain information on this point.

It has been said, that this may be attributed to the fact that ether is not extensively used, but that if it were, there would probably have been as many fatal cases in proportion from it, as from the inhalation of chloroform. But this statment is not strictly correct; for though ether is not employed as an anæsthetic agent to any extent, if at all, in Great Britain or many parts of Europe, it is used in Lyons, Naples, and is almost the only one that is administered in the principal hospitals of the United States of America, where its now familiar properties were first discovered.

I have given it in several hundred cases, and witnessed its exhibition by others in as many more. I have administered it to infants not three weeks old, and to persons more than three score years and ten, and have never in a single instance seen an alarming or distressing effect produced by it. On the first introduction of ether into surgical practice, it was not thought safe to allow persons to inhale it in whom there was reason to believe there was any disease of the heart or lungs, or who had any tendency to an affection of the brain and nervous system. But for some years past I have been in the habit of administering it to individuals of this description, and have as yet had no cause to regret it. In such cases I have thought it prudent to have the vapor of the ether inhaled more slowly, so that it may be more diluted with atmospheric air than under ordinary circumstances; of course, the patient could not be brought as soon under its influence as when taken in the ordinary way.

The state of the system which is produced by the inhalation of ether, is that of narcotism, similar precisely to what is induced by drinking immoderately wine or other alcoholic liquors. It is a state of intoxication more transient and less dangerous than that from alcohol. Its effects pass off sooner, because the vapor of the ether begins to escape from the lungs as soon as the patient ceases to inhale it; while alcohol taken into the stomach is carried into the circulation, and mixes with the blood, and in this way acts longer, if not more powerfully on the brain, though its narcotic effect is not so soon produced. It is possible that life might be destroyed by the inhalation of ether, if it be continued uninterruptedly for a great length of time and a great quantity inhaled. Fatal congestion of the brain might thus be produced, as sometimes happens when alchoholic liquor has been taken to excess. But no person of ordinary prudence would administer it in this way. Long before the occurrence of such a result, symptoms of an unequivocal character would indicate the approaching danger.

When death follows the inhalation of chloroform, on the other hand, there is no merciful premonition. The late Dr. Snow, whose experience on the subject was perhaps greater than that of any other person, thought that "*sudden palsy of the heart* is the cause of sudden death from chloroform." In death by asphyxia, the heart beats for some minutes after breathing has ceased; "whereas in some cases of death by chloroform, the breathing has been proved to go on up to the time the pulse stopped, and after it."

With the hope that those who may have occasion to employ any anæsthetic agent will at least make a fair trial of *rectified sulphuric ether*, I respectfully submit these remarks to my professional brethren.—*Brit. and For. Med. Chir. Review, Oct.* 1859, *p.* 484.

---

### ACUTE PERIOSTITIS.

By T. B. Curling, Esq., Surgeon to the London Hospital.

There are few operations more frequently performed in the hospitals of London than those required for the removal of necrosed bone, and they are certainly more common now than they were in former years. The results show that these operations are essentially of a conservative character,—that tedious sinuses, always discharging and liable to inflame, have been enabled to close, and that useful limbs have been saved by the removal of a constant source of irritation. These operations have become more frequent, then, because surgeons have become more strongly impressed with the advantage of extracting locked-up or impacted dead bone—have become less afraid of ill-consequences from the necessary disturbance of parts, and have been emboldened to undertake tedious and troublesome operations, knowing that they can be rendered painless by chloroform. Our museums are rich in specimens of encased bone taken from amputated limbs; but such preparations are rarely added now, because it is seldom that a limb is removed for such a disease until the attempt to save it has been made by extracting the incarcerated bone. But so numerous are cases of necrosis in hospital practice that it is well to inquire whether we can not, in many instances, prevent this serious result. The more common cause of necrosis in the long bones is acute periostitis; consequent upon injury.

When we consider that the whole, or greater part of a bone may be destroyed by periostitis—that the inflammation may extend to the adjoining articulations, imperiling the safety of a limb, and that patients sometimes sink under the constitutional fever attending it, I need not urge the importance of an early diagnosis of the disease, in order that right and prompt measures may be taken for its removal. The complaint for which acute periostitis is most liable to be mistaken is acute rheumatism; and it is a mistake which, I fear, is not unfrequently made in practice. Indeed, some care and nice observation are required to make the diagnosis. In rheumatism, as in periostitis, there is high inflammatory fever, with swelling of the limb, and great pain, increased by pressure, so that the patient is nearly helpless and he shrinks from the touch of the surgeon in dread of the torture which an examination may cause him. In periostitis, say of the femur or tibia, the swelling is diffused. It is not limited to the larger joints—to the ankle or to the knee, but occupies a wider range, and is œdematous in character. But the chief diagnostic mark is the seat of pain. In periostitis little or no pain is caused by pressure, unless it be made over, or in the course of, the affected bone. You may, in the early stage, move the limb at the knee or the ankle, and press the ligaments and tendons without producing pain, but the slightest pressure on the bone excites intense suffering. If you press over the tibia or the muscles of the thigh around the femur in rheumatism, you rarely cause much pain; but in acute periostitis such pressure can not be tolerated for a moment. The conclusion in favor of periostitis will be much strengthened if it be found that the attack of inflammation succeeded an injury.

The treatment commonly recommended in acute periostitis is local depletion with calomel and opium. Just at the onset of an attack, in a superficial bone like the tibia, this treatment may be of service, but in periostitis of a deep-seated bone, or if the inflammation do not speedily subside, such measures are not to be relied on. After matter has formed beneath the membrane, they are worse than useless. They weaken the patient without exerting any influence on the disease. There is then no way of averting serious mischief but by a free incision of the inflamed periosteum.—*Lancet, Sept.* 3, 1859, *p.* 231.

---

The Grape Treatment on the Teeth.—A writer in the *Lancet* says that the grape cure exerts a deleterious influence on the teeth. He has seen the front incisors of a patient greatly corroded after spending some time under the treatment at Vevey.

### DEFECTIVE ASSIMILATION IN INFANTS—ITS PREVENTION AND TREATMENT.

BY DR. ROUTH.

[The greater part of the mortality of infants is due to the defective assimilation—the result of want of breast-milk, and the use of injudicious food. Diseases of defective assimilation are favored by hereditary tubercular habit, exanthemata, bad air, and want of cleanliness. After death when diarrhœa has been present, red patches or aptha are found on the alimentary mucous membrane, or a reddish-colored intensely acid mucus is found exuded from it.]

The disease seems to be gradual, passing on to entire loss of *primary* assimilation; the secondary still persisting, although inactive from want of assimilable matters to take up. Albuminous, starchy, and oily matters were not digested.

The *treatment* consists in supplying fatty acids and already artificially digested animal and occasionally vegetable substances, especially human milk. If this could not be sucked, it should be collected in a cup and given by the spoon. Dr. Routh strongly animadverted here upon the absurd dogma, that it is wrong to mix human and cow's milk. He, on the contrary, believed the plan not only safe, but the very best practice in many cases, and the only means of saving an infant's life. Simple juice of meat, and this with vegeto-animal food, he had found most useful in fulfilling these indications. The remedies were of two kinds: 1st. Those calculated to increase cell growth and development. Phosphate of soda, producing an emulsion with fats, thus allowing of their assimilation; chloride of potassium, to dissolve carbonate of lime; phosphate of lime, to enable blood to take up more carbonic acid, and thus hold in solution more carbonate of lime; (these substances severally strengthening muscular and bony structure;) lime-water to provide lime to blood. 2d. These last also acted as some of the remedies calculated to allay local irritation of the alimentary canal. Carminatives were useful, such as dill, but especially cinnamon-powder, to correct flatus and to check diarrhœa. Anodynes were also (however objected to generally) strongly recommended by the author. For the diarrhœa, when present, nitrate of silver, and sulphate of copper were the best remedies. Wine was also found very serviceable, even if given in large quantities. These remedies, however, it must be confessed, proved in most cases of no avail in the third stage, which was, he might say, almost incurable; but they acted very effectively in the second and first stages.—*Lancet, June* 18, 1859, *p.* 613.

---

Fatality from Chloroform.—Dr. Kidd, who has given much attention to the subject of chloroform, has observed that deaths attributable to its inhalation have occurred more frequently during the performance of the minor surgical operations. The statistics of deaths from chloroform certainly show a much greater proportion in the performance of trifling operations, as of 85 fatal cases in which the nature of the operations was recorded, 10 were extractions of teeth, 14 removals of toe-nails and operations on phalanges, while of this number none occurred in the performance of the large amputations, resection or ligature of large arteries, etc.

Dr. Kidd has therefore hastily concluded from these results, that "chloroform is safer in large than in small operations." He seems to have overlooked the fact of the vastly greater frequency of the performance of small operations, and of course the more frequent administration of the anæsthetic, which is, we believe, sufficient to account for the apparent greater fatality attending minor operations.

Dr. Kidd estimates the number of deaths from inhalation of chloroform to be about one hundred. We think that if this number had been quadrupled it would more nearly approximate the truth. Chloroform never gained general confidence in this country, and its use has within the past few years rapidly declined, yet the deaths referable to it would probably equal one half of Dr. Kidd's entire estimate of fatality from it.

The European origin of chloroform inhalation and its distinguished authorship, has given it a confidence which can not long be maintained in the face of such uncontrollable mortality, and while the causes of sudden death from it are so little understood.—*Medical and Surgical Reporter.*

---

Salivary Calculus.—J. H., aged 48 years, of spare habit and slender constitution, some fourteen years since was seized with a severe pain under the left side of his tongue.

He applied to his family physician, who could give him no satisfactory information as to the cause or nature of his complaint; neither could he afford him any relief. He was induced to consult other physicians in his vicinity, and he did so with like results. In the meantime a small tumor made its appearance on the under side of his tongue, near or at the seat of pain. He went to Boston and consulted the late Dr. ——, who informed him that his disease was cancer, and gave him but little encouragement as to any permanent relief. He returned to his home, determined to abide the result of what he then supposed an incurable disease. From that time until about the first of February last, he has suffered paroxysms of severe and excruciating pain at different times. The tumor gradually increased in size, and the paroxysms of pain became more frequent, until it finally became inflamed, supperated and burst, discharging a small quantity of pus and a calculus weighing fifteen grains, having the general appearances of ordinary renal or billiary calculi. He has since been entirely free from pain.—*Communicated for the Boston Medical and Surgical Journal.*

TESTS FOR THE PURITY OF CHLOROFORM.—M. Berthe gives the following directions in the "Moniteur des Hopitaux": Chloroform may contain chloride of elaidine, alcohol, various chlorides, amylic, and methylic combinations, and aldehyde. By adding caustic potash to chloroform, containing chloride of elaidine, the compound is transferred into chloride of acetyle, the fœtor of which is immediately noticed. In order to ascertain the presence of all the other compounds which may be mixed with the chloroform, especially alcoholic compounds, pound a small quantity of bichromate of potash in a little chloroform, and add to this mixture a few drops of sulphuric acid. If the chloroform is pure, a reddish-brown precipitate of chromic acid is formed; if not pure, the acid is reduced, whilst the precipitate, or sometimes the liquid itself, assumes a green color, dependent on the presence of the sesqui-oxide of chrome.—*Lancet, Aug.* 27, 1859, *p.* 218.

BLOODLESS OPERATION FOR CLEFT PALATE.—It may be recollected that M. Jules Cloquet published, in 1855, an essay on the method of applying cauterization to the abnormal cleavage of certain organs, and that cases were therein mentioned in which union of the margins of the cleft had been obtained by repeated cauterization. At the meeting of the Academy of Science of Paris of the 21st May, M. Cloquet brought forward a case treated by Prof. Benoit, of Montpelier. The child was eleven years old; the soft palate was completely cleft, and all the usual symptoms were present. The treatment lasted nineteen months, with two rather long interruptions. The whole cleft has now united, save that of the uvula, and this result was obtained by 33 cauterizations, 14 with the acid nitrate of mercury, and 19 with the solid nitrate of silver. A slight nasal pronunciation still exists, being the result of habit. M. Benoit means to apply the same treatment to the uvula.—*Lancet.*

Sulphuric Ether as a Substitute for Chloroform.—The Medical Times & Gazette states, that at Lyons, France, ether has almost universally superseded chloroform as an anæsthetic, both in hospitals and private practice. After a discussion of the subject by the Medical Society of that town, resolutions were adopted to the following effect: 1st Sulphuric ether employed as an anæsthetic is less dangerous than chloroform, no accident having followed its exclusive and abundant employment at Lyons during eight years; 2d. Anæsthesia may be as abundantly and completely induced by it as chloroform; 3d. Ether should, therefore, be preferred to chloroform. This but confirms the experience of medical men in this country on the same subject.

Death from the Inhalation of Chloroform.—Fatal cases from the inhalation of chloroform are multiplying. Two deaths from this cause have occurred in Bellevue Hospital, N. Y., in less than a year, and others are reported elsewhere until we have grown weary of recording them. The question is raised, and should be seriously considered, whether sulphuric ether, which is said to be entirely safe, should not be substituted for chloroform, notwithstanding its slowness of action?

The following letter on the use and abuse of tobacco, present some of the points so well that we can not refrain from giving it a place in the Register.

Upon this subject of tobacco there is an exceeding diversity of opinion, arising from the different effects manifested by it, upon different individuals; and this resulting from the different susceptibilities, and the different modes in which it is used. Upon these two points turns all the variety of manifestation exhibited in the use of this article. Some use it sparingly, so that no palpable systematic effect is produced; while others use it to such excess that the system becomes saturated with it, so that not only is the breath loaded with its odor, but every excretion of the body is tinctured, and in some cases saturated with it.

There is a great diversity of susceptibility to the influence of tobacco ; some will use large quantities without seeming to be very seriously affected by it, while in others, even a small quantity will produce very marked effects. But aside from all this, we have a few words in regard to its use by dentists. The use of tobacco in any ordinary way, by the dentist, is a practice which we consider reprehensible, in point of propriety and etiquette. It is in all cases offensive and disgusting in proportion to the carelessness with which it is used, and the amount. Some are so careless, that in using a small amount they are very offensive.

No one can use even a small amount without the breath becoming tainted with it, and this to many persons is sickishly offensive ; how a dentist of any refinement can persist in such an infliction upon his patients, is more than we can comprehend. But we intend in future to say something more upon this subject.—Ed.

The Use and Abuse of Tobacco.—*Sir* :—Having been applied to some time since to join in a petition to the House of Commons that they would appoint a committee to inquire into the effects produced by the prevailing habit of tobacco smoking, I declined to do so ; first, because it did not appear to me that such a committee would be very competent to discuss a question of this kind; and secondly, because, even if they were so, I did not see that it would be possible for Parliament to follow up by any act of legislation the conclusions at which they might have arrived. Nevertheless I am ready to admit that the subject is one of no trifling importance, and well worthy the serious consideration of any one who takes an interest in the present and future well-being of society. From these considerations it is that I now venture to address to you the following observations.

The empyreumatic oil of tobacco is produced by distillation of that herb at a temperature above that of boiling water. One or two drops of this oil (according to the size of the animal) placed on the tongue will kill a cat in the course of a few minutes. A certain quantity of the oil must be always circulating in the blood of an habitual smoker, and we can not suppose that the effects of it upon the system can be merely negative. Still, I am not prepared to subscribe to the opinions of those who hold that, under all circumstances, and to however moderate an extent it be practiced, the smoking of tobacco is prejudicial. The first effect of it is to soothe and tranquilize the nervous system. It allays the pains of hunger and relieves the uneasy feelings produced by mental and bodily exhaustion. To the soldier who has passed the nignt in the trenches before a beleagured town, with only a distant prospect of breakfast when the morning has arrived; to the sailor, contending with the elements in a storm; to the laborer, after a hard day's work; to the traveler in an uncultivated region, with an insufficient supply of food, the use of a cigar or a tobacco pipe may be not only a grateful indulgence, but really beneficial. But the occasional use of it under such circumstances is a very different matter from the habit of constant smoking which prevails in certain classes of society at the present day.

The effects of this habit are, indeed, various, the difference of constitution, and difference in the mode of life otherwise. But, from the best observations which I have been able to make on the subject, I am led to believe that there are very few who do not suffer harm from it, to a greater or less extent. The earliest symptoms are manifested in the derangement of the nervous system. A large proportion of habitual smokers are rendered lazy and listless, indisposed to bodily and incapable of much mental exertion. Others suffer from depression of the spirits, amounting to hypochondriasis, which smoking relieves for a time, though it aggravates the evil afterwards. Occasionally there is a general nervous excitability, which, though very much less in degree, partakes of the nature of the *delirium tremens* of drunkards. I have known many individuals to suffer from severe nervous pains, sometimes in one, sometimes in another part of the body. Almost the worst case of neuralgia that ever came under my observation was that of a gentleman who consulted the late Dr. Bright and myself. The pains were universal, and never absent; but during the night they were especially intense, so as almost wholly to prevent sleep. Neither the patient himself nor his medical attendant had any doubts that the disease was to be attributed to his former habit of smoking, on the discontinuance of which he slowly and gradually recovered. An eminent surgeon, who has a great experience in ophthalmic diseases, believes that, in some instances, he has been able to trace blindness from amaurosis to excess in tobacco smoking; the connection of the two being pretty well established in one case by the fact that, on the practice being left off, the sight of the patient was gradually restored. It would be easy for me to refer to other symptoms indicating deficient power of the nervous system to which smokers are liable; but it is unnecessary for me to do so; and, indeed, there are some which I would rather leave them to imagine for themselves than undertake the description of them myself in writing.

But the ill effects of tobacco are not confined to the nervous system. In many instances there is a loss of the healthy appetite for food, the imperfect state of the digestion being soon rendered manifest by the loss of flesh and the sallow countenance. It is difficult to say what other diseases may not follow the imperfect assimilation of food contained during a long period of time. So many causes are in operation in the human body which may tend in a greater or less degree to the production of organic changes in it, that it is only in some instances we can venture to pronounce as to the precise manner in which a disease that proves mortal has originated. From cases, however, which have fallen under my own observation, and from a consideration of all the circumstances, I can not entertain a doubt that, if we could obtain accurate statistics on the subject, we should find that the value of life in inveterate smokers is considerably below the average. Nor is this opinion in any degree contradicted by the fact that there are individuals who, in spite of the inhalation of tobacco smoke, live to be old, and without any material derangement of the health ; analagous exceptions to the general rule being met with in the case of those who have indulged too freely in the use of spirituous and fermented liquors.

In the early part of the present century tobacco smoking was almost wholly confined to what are commonly called the lower grades of society. It was only every now and then that any one who wished to be considered as a gentleman was addicted to it. But since the war on the Spanish Peninsula, and the consequent substitution of the cigar for the tobacco-pipe, the case has been entirely altered. The greatest smokers at the present time are to be found, not among those who live by their bodily labor, but among those who are more advantageously situated, who have better opportunities of education, and of whom we have a right to expect that they should constitute the most intelligent members of the community. Nor is the practice confined to grown-up men. Boys, even at the best schools, get the habit of smoking, because they think it manly and fashionable to do so ; not unfrequently because they have the example set them by their tutors, and partly because there is no friendly voice to warn them as to the special ill consequences to which it may give rise where the process of growth is not yet completed, and the organs are not yet fully developed.

The foregoing observations relate to the habit of smoking as it exists among us at the present time. But a still graver question remains to be considered. What will be the result if this habit be continued by future generations? It is but too true that the sins of the fathers are visited upon their children and their children's children. We may here take warning from the fate of the red Indians of America. An intelligent American physician gives the following explanation of the gradual extinction of this remarkable people: One generation of them become addicted to the use of the fire-water. They have a degenerate and comparatively imbecile progeny, who indulge in the same vicious habit with their parents. Their progeny is still more degenerate, and after a very few generations the race ceases altogether. We may also take warning from the history of another nation, who some few centuries ago, while following the banners of Solyman the Magnificent, were the terror of Christendom, but who since then, having become more addicted to tobacco smoking than any of the European nations, are now the lazy and lethargic Turks, held in contempt by all civilized communities.

In thus placing together the consequences of intemperance in the use of alcohol and that in the use of tobacco, I should be sorry to be misunderstood as regarding these two kinds of intemperance to be in an equal degree pernicious and degrading.

The inveterate tobacco-smoker may be stupid and lazy, and the habit to which he is addicted may gradually tend to shorten his life and deteriorate his offspring, but the dram drinker is quarrelsome, mischievous, and often criminal. It is under the influence of gin that the burglar and the murderer become fitted for the task which they have undertaken. The best thing that can be said for dram-drinking is, that it induces disease which carries the poor wretch prematurely to the grave, and rids the world of the nuisance. But unfortunately in this, as in many other cases, what is wanting in quality is made up in quantity. There are checks on one of these evil habits which there are not on the other. The dram-drinker, or, to use a more general term, the drunkard, is held to be a noxious animal. He is an outcast from all decent society, while there is no such exclusion for the most assiduous smoker.

The comparison of the effects of tobacco with those of alcohol leads to the consideration of a much wider question than that which I set out. In all ages of which we have any record, mankind have been in the habit of resorting to the use of certain vegetable productions, not as contributing to nourishment, but on account of their having some peculiar influence as stimulants or sedatives (or in some other way) on the nervous system. Tobacco, alcohol, the Indian hemp, the kava of the South Sea Islanders, the Paraguay tea, coffee, and even tea, belong to this category. A disposition so universal may almost be regarded as an instinct, and there is sufficient reason to believe that, within certain limits, the indulgence of the instinct is useful. But we must not abuse our instincts. This is one of the most important rules which man, as a responsible being, both for his own sake, and for that of others, is bound to observe. Even such moderate agents as tea and coffee, taken in excess, are prejudicial. How much more so are tobacco and alcohol, tending, as they do, not only to degradation of the individual, but that of future generations of our species.

If tobacco-smokers would limit themselves to the occasional indulgence of their appetite, they would do little harm either to themselves or others; but there is always danger that a sensual habit once begun may be carried to excess, and that danger is never so great as in the case of those who are not compelled by the necessities of their situation to be actively employed. For such persons the prudent course is to abstain from smoking altogether.

Trusting that you and your readers will excuse me for having occupied so large a space in your columns,

I am, Sir, your obedient servant,

Augus 27. B. C. BRODIE.

*Med. Timəs and Gaz.*, Sept. 8th, 1860.

---

STILL ANOTHER DEATH FROM CHLOROFORM—SHALL ITS USE CONTINUE?—A death from chloroform, at the Northampton Infirmary, is recorded in the London *Medical Times and Gazette.* The patient was about to submit to an operation for the removal of a small tumor from the back. Chloroform was administered cautiously. The testimony before the coroner's jury says that the effects of the anæsthetic were soon visible upon the deceased, who became insensible without anything unusual being observed, although he was closely watched. On removing him into a proper position for performing the operation, it was observed that his countenance was very much changed. The suspicions of the operators were at once roused, and immediate steps were adopted for bringing the man to his senses again, instead of commencing the surgical operation. Restoratives were resorted to, but to no purpose. Artificial respiration was then attempted, but this, too, was unavailing, and after an hour's futile endeavors at respiration, the deceased was reluctantly given up as lost.

We record this case, not from any peculiar interest that it possesses, but that it may be added to the long, dark list which now stands against chloroform. All statistics of fatalities from chloroform which we have seen, are inaccurate, and far below the truth in their estimates, and we desire that every fatal case attributable to it may hereafter be presented. It is hoped that such cases, instead of being concealed, as they often have been, as if the unfortunate administrator felt guilty of homicide, will henceforth be published. We believe that, had all the deaths from chloroform been properly noted, a list could now be presented which would astonish most of the advocates of its use, and do much in future to prevent loss of life by causing the discontinuance of its administration.

The practice of administering chloroform is rapidly decreasing in this country, and we sometime ago predicted its discontinuance for general anæsthetic purposes, and the substitution of ether. If its use is not lessening in Europe, there is a growing want of confidence in its safety. The very caution with which European surgeons give it, shows that they use it with a consciousness of its danger. In the testimony of this case it was stated that the operator had the precaution to examine the deceased, "to ascertain if he was able to bear the effect of the chloroform." In the use of ether, which is now admitted to be almost absolutely safe, no one ever thinks of using such precautions, and it is now deemed admissible in any condition in which its anæsthetic effects are desirable.

We relinquished the convenient use of chloroform with reluctance. When deaths under its administration became frequent, we still hoped that greater caution in its use, increased study of its physiological effects, further knowledge of the conditions which contra-indicate it, and a discovery of the constitutional idiosyncrasies which make the administration fatal, would enable us to continue its use with safety. But the fatalities are now as numerous, as unexpected, and as inexplicable as ever. Patients in vigorous health and under the most cautious hands, continue to die when but little of the vapor has been inhaled, and sometimes at almost the first inspiration.

In the present state of our knowledge of the mysterious fatal influences of chloroform, and its acknowledged uncontrollable mortality, we consider its ordinary use unjustifiable while an efficient and safe alternative for use is at hand. If the profession do not discontinue its use, patients will soon refuse it. There is already, owing to its dangers, an increasing prejudice among the masses in regard to anæsthesia, in whatever manner produced, and if the use of chloroform, with its fatal accompaniments, continues, the popular verdict will condemn anæsthetics entirely, preferring to suffer pain rather than incur such a hazard of life.—*Medical and Surgical Reporter.*

Professional Maxims.—The difficulties and the honors of professional life, the value of labor, and the failures of genius, are subjects which can never grow trite and wearisome to the mind of the professional reader. Few men will read without interest the words which Lord Stanley addressed to the students of University College in presenting the successful candidates with their hardly-earned prizes. He told them that real work—work which will stand the test of time and the criticism of competent judges—can never be the result of hasty, careless, or desultory efforts. The rapid intuition, the sweeping glance, the ready analysis, and the triumphant argument, in which we admire the skillfulness of a born genius, are the evidences of trained thought and well-drilled intelligence, of which the schooling has often been secretly accomplished. So that facility becomes a measure for previous labor, and readiness an index of early preparation. The students were warned, too, against an error which many hundreds of members of the medical profession must now deplore. They were told that when once a man has thrown himself heart and soul into the turmoil and anxiety of professional life, there is not for him much more—in ninety-nine cases out of a hundred there is no more—of that free and liberalizing study which develops not one faculty, but all the faculties of the mind. There is an universal and almost irresistible impatience to burst the bonds of college discipline, and to spring into active life. The phrase is—to waste no time but to buckle to the work of life at once. But time spent in study, while the habit of mental application is yet strong and the mind vigorous and impressible, is not lost; it gives such a perfection of mental culture as heightens all the powers, and helps at once to happiness and success. Wisely did Lord Stanley say to these young men—"Study as long as in prudence you can, and don't fear that life will not be long enough to reap what you have sown." It is almost to be regreted that any are permitted to engage in the active pursuits of life, as in our profession, when just of age. The young man of twenty-one, who will give three years to study and mental cultivation, will always look back upon the time so spent with pleasurable satisfaction.—*Lancet.*

